# Human Milk, Protein Adequacy, and Neurodevelopment in Preterm Infants: A Narrative Review

**DOI:** 10.3390/children13101317

**Published:** 2026-09-27

**Authors:** Katsumi Mizuno

**Affiliations:** Department of Pediatrics, Showa Medical University, Tokyo 142-8666, Japan; katsuorobi@med.showa-u.ac.jp

**Keywords:** human milk, preterm infant, neurodevelopment, multi-organ protection, human milk-derived fortifier

## Abstract

**Highlights:**

**What are the main findings?**

- Human milk is a biologically active nutritional exposure that supports multiple developmental pathways in very preterm infants.
- Mother’s own milk is associated with favorable neurodevelopmental and neu-roimaging outcomes, although much of the evidence remains observational.
- Nutritional adequacy is essential because unfortified human milk alone generally does not meet the protein, energy, mineral, and micronutrient requirements of very preterm infants.

**What are the implications of the main findings?**

- Fortification strategies should be individualized according to milk source, nutrient intake, growth, feeding tolerance, metabolic status, and clinical context.
- An exclusive human milk diet is a biologically plausible and clinically promising strategy, but whether its short-term nutritional and clinical benefits translate into sustained neurodevelopmental benefits requires further study.

**Abstract:**

Human milk is increasingly recognized not only as a source of nutrition but also as a biological regulator of organ development in preterm infants. Beyond supporting growth, human milk contains a complex array of bioactive components that influence intestinal maturation, immune function, vascular development, and brain development during a critical period of extrauterine adaptation. This narrative review summarizes current evidence regarding the role of human milk in multi-organ development in preterm infants. We review clinical and mechanistic studies demonstrating associations between human milk exposure and reduced risks of necrotizing enterocolitis, late-onset sepsis, bronchopulmonary dysplasia, and retinopathy of prematurity. Particular attention is given to the interconnected gut–immune–lung–retina–brain axis and the biological pathways through which human milk may influence developmental outcomes. We further discuss the importance of early enteral feeding, adequate protein delivery, and human milk-derived fortification in supporting growth quality, lean mass accretion, head growth, and structural brain development. Emerging evidence suggests that nutritional strategies promoting early and sustained human milk exposure may contribute not only to protection from major prematurity-related morbidities but also to improved neurodevelopmental trajectories. By integrating current biological and clinical evidence, this review highlights the role of human milk as a foundation for developmental nutrition in preterm infants and provides a practical perspective for optimizing nutritional care in the neonatal intensive care unit.

## 1. Introduction

Survival of very preterm infants (VPIs) has improved substantially over recent decades owing to advances in respiratory care, infection control, and neonatal intensive care practices. However, major prematurity-related morbidities, including necrotizing enterocolitis (NEC), bronchopulmonary dysplasia (BPD), retinopathy of prematurity (ROP), late-onset sepsis (LOS), postnatal growth failure, and neurodevelopmental impairment, remain important challenges. These conditions frequently arise during the early postnatal period and may have long-term consequences for growth, health, and neurodevelopment.

Nutrition is one of the few modifiable components of neonatal care that may influence both short- and long-term outcomes in preterm infants. During the third trimester, rapid growth and maturation occur across multiple organ systems, including the intestine, immune system, lung, retina, and brain. Preterm birth abruptly interrupts this developmental process and shifts a substantial part of fetal growth and organ maturation to the neonatal intensive care unit (NICU), where achieving adequate nutrient delivery while minimizing feeding-related complications represents a major clinical challenge.

Human milk (HM) is widely recognized as the preferred source of enteral nutrition for preterm infants [1,2]. In addition to providing essential nutrients, HM contains numerous bioactive components, including human milk oligosaccharides (HMOs), lactoferrin, immunoglobulins, growth factors, antioxidants, and bioactive lipids [3]. These components may contribute to intestinal maturation, immune regulation, microbial colonization, vascular development, and brain development. Human milk therefore represents not only a source of nutrients but also a biologically active nutritional exposure during a critical period of extrauterine development (Table 1).

Accumulating evidence indicates that greater HM exposure is associated with lower risks of NEC [16,17,18], BPD [19,20,21,22], ROP [23,24], and LOS [25], as well as lower overall morbidity in very low-birth-weight infants [26]. Greater HM exposure, particularly mother’s own milk (MOM), has also been associated with more favorable neurodevelopmental and neuroimaging outcomes [27,28,29,30]. However, much of the evidence relating HM exposure to neurodevelopment is observational, and the strength of these associations varies according to milk source, dose and duration of exposure, nutritional adequacy, clinical characteristics, and study design. Accordingly, associations observed for MOM should not automatically be extrapolated to pasteurized donor human milk (DHM), and effects associated with an exclusive human milk diet (EHMD) should not necessarily be attributed to human milk-derived fortifier (HMDF) alone.

This distinction is particularly important in VPIs because the biological advantages of HM do not necessarily ensure nutritional adequacy. Unfortified HM may not provide sufficient protein, energy, minerals, and micronutrients to meet the requirements of rapidly growing preterm infants. Thus, the potential developmental effects of HM need to be considered together with the adequacy of nutrient delivery, the source and timing of fortification, growth and body composition, and the occurrence of neonatal morbidities that may themselves influence later neurodevelopment.

Breastfeeding is associated with important child and maternal health benefits. Large-scale epidemiological analyses have estimated that optimal breastfeeding practices could prevent approximately 823,000 child deaths annually worldwide, largely through reductions in infectious morbidity and mortality [31]. In preterm infants, however, interpretation of the evidence requires additional consideration of milk source, processing, nutrient composition, fortification, and the clinical context in which HM is provided. Mother’s own milk, DHM, unfortified HM, fortified HM, HMDF, cow’s milk-derived fortifier (CMDF), and an EHMD therefore represent related but distinct nutritional exposures and should not be considered interchangeable.

Many previous reviews have focused primarily on individual aspects of human milk feeding, such as milk source, fortification, growth, neonatal morbidity, or neurodevelopment. The present review integrates these domains within a developmental nutrition framework linking milk source, nutrient and protein adequacy, fortification strategy, growth and body composition, neonatal morbidity, brain development, and later neurodevelopment. Central to this framework is the concept that the biological properties of HM and the nutritional adequacy of the overall diet are complementary but distinct considerations in the nutritional care of VPIs.

Accordingly, the aim of this structured narrative review is to critically synthesize current evidence regarding HM-based nutrition, protein adequacy, fortification strategies, growth, neonatal morbidity, brain development, and neurodevelopment in preterm infants. We distinguish MOM, DHM, unfortified and fortified HM, CMDF, HMDF, and an EHMD; interpret clinical and mechanistic evidence according to study design and evidentiary strength; and consider both direct nutritional pathways and indirect pathways involving growth and major prematurity-related morbidities. By integrating these dimensions, this review aims to clarify what is supported by current evidence, where causal inference remains limited, and which knowledge gaps should be prioritized in future studies of developmental nutrition in VPIs.

## 2. Methods of the Narrative Review

This review was conducted as a structured narrative review to critically examine the relationships among human milk exposure, protein adequacy, fortification strategies, growth, brain development, neonatal morbidity, and neurodevelopmental outcomes in preterm infants. A literature search was conducted in PubMed for English-language articles published through August 2026. Search terms included combinations of “preterm infant,” “very low birth weight,” “human milk,” “mother’s own milk,” “donor human milk,” “human milk fortifier,” “exclusive human milk diet,” “protein intake,” “growth,” “body composition,” “brain development,” “magnetic resonance imaging,” and “neurodevelopment.” Reference lists of relevant reviews, systematic reviews, and primary studies were also examined to identify additional publications.

Studies were considered eligible when they included preterm or very low-birth-weight infants and evaluated one or more of the following domains: human milk source or exposure, fortification strategy, protein or energy intake, growth or body composition, neonatal morbidity, brain imaging, or neurodevelopmental outcomes. Studies conducted exclusively in term infants were generally not included in the clinical evidence synthesis. However, mechanistic, preclinical, and studies of individual human milk components were considered when they provided relevant information regarding biological plausibility.

Priority was given to systematic reviews, meta-analyses, randomized controlled trials, prospective cohort studies, and studies reporting neurodevelopmental or neuroimaging outcomes. Observational studies were included when randomized evidence was unavailable or when they provided information on dose–response relationships, long-term outcomes, or clinically relevant nutritional exposures. Mechanistic and preclinical evidence was used to support biological plausibility and was not interpreted as evidence of clinical efficacy.

Potentially relevant publications were assessed according to their relevance to the predefined themes of this review. Evidence was synthesized thematically according to human milk source, nutrient and protein adequacy, fortification strategy, growth and body composition, neonatal morbidity, brain development, and neurodevelopmental outcomes. Greater interpretive weight was given to systematic reviews, meta-analyses, randomized controlled trials, and prospective studies, whereas observational and mechanistic studies were used primarily to address questions for which higher-level evidence was limited.

Because this was a narrative rather than a systematic review, study selection, data extraction, and risk-of-bias assessment were not conducted according to a prespecified systematic-review protocol. The findings were interpreted according to study design and evidentiary strength, consistent with the emphasis on scientific reasoning and appropriate presentation of evidence in the Scale for the Assessment of Narrative Review Articles (SANRA) [32]. Particular attention was paid to distinguishing MOM, DHM, unfortified HM, CMDF, HMDF, and an EHMD, because these exposures represent different nutritional interventions and should not be considered interchangeable.

## 3. Defining Human Milk-Based Nutritional Exposures

Interpretation of studies examining human milk and neurodevelopment requires careful distinction among the nutritional exposures being evaluated. MOM, DHM, unfortified HM, fortified HM, and an EHMD are not equivalent interventions. Their nutritional composition, bioactive properties, clinical indications, and supporting evidence differ substantially [1,33]. Studies that combine these exposures under the general term “human milk feeding” may therefore obscure clinically important differences.

### 3.1. Mother’s Own Milk and Donor Human Milk

MOM is the preferred source of enteral nutrition for preterm infants because it provides species-specific nutrients and bioactive components and adapts, at least partly, to the infant’s gestational age and stage of lactation [1,34]. Greater exposure to MOM during neonatal hospitalization has been associated with more favorable cognitive, motor, and neuroimaging outcomes in several cohort studies [28,29,30]. However, most of this evidence is observational and may be influenced by maternal education, socioeconomic circumstances, illness severity, parental involvement, and other factors associated with both MOM availability and later development [1,29].

When MOM is unavailable or insufficient, pasteurized DHM is generally preferred to preterm formula, particularly for infants at high risk of NEC [1,35]. However, DHM should not be assumed to have the same nutritional or developmental effects as MOM. DHM is commonly obtained from mothers who delivered at term and are at a later stage of lactation, and its protein content may therefore be lower than that of early preterm MOM. Collection, storage, freezing, and Holder pasteurization may additionally alter the activity of some cellular, enzymatic, and immunological components [1,33,34]. These factors may reduce protein content and the activity of some cellular, enzymatic, and immunological components. Although DHM provides an important means of avoiding or reducing formula exposure, randomized trial evidence has not demonstrated a clear long-term neurodevelopmental advantage over formula [35]. Findings concerning MOM and neurodevelopment should therefore not be extrapolated automatically to DHM.

### 3.2. Unfortified and Fortified Human Milk

Unfortified HM does not generally provide sufficient protein, minerals, and energy to meet the requirements of very preterm infants at customary feeding volumes [1,33]. This distinction is essential when interpreting associations between HM feeding, postnatal growth, and neurodevelopment. A high proportion of HM exposure does not necessarily indicate adequate nutrient intake, particularly when the milk composition has not been measured and fortification practices are insufficient or inconsistently reported [1].

HM may be fortified with either CMDFs or HMDFs. CMDFs are widely used and increase the protein, energy, mineral, and micronutrient content of MOM or DHM. HMDFs are derived from donated HM and permit fortification without the introduction of cow’s milk-derived protein. Both approaches are intended to address the nutritional limitations of unfortified HM, but they differ in composition, processing, cost, availability, and supporting evidence [1,36].

Fortification may be implemented using standard, adjustable, or targeted approaches. Standard fortification provides a fixed amount of fortifier based on an assumed milk composition, whereas adjustable fortification modifies protein delivery according to biochemical markers, such as blood urea nitrogen. Targeted fortification uses measured macronutrient composition to individualize nutrient supplementation. Because these strategies can result in different actual protein and energy intakes, studies reporting only that HM was “fortified” may not adequately characterize the nutritional exposure.

### 3.3. Exclusive Human Milk Diet

An EHMD generally refers to a feeding strategy in which all enteral nutrition is derived from HM, including MOM, DHM when supplementation is required, and a HMDF. Thus, an EHMD is a composite nutritional strategy rather than a single product or intervention. Its observed effects cannot necessarily be attributed solely to the fortifier because studies may simultaneously differ in the proportions of MOM and DHM, timing of formula exposure, feeding advancement, fortification thresholds, and nutrient intake.

Some studies have compared an EHMD with feeding strategies that include CMDF or preterm formula. These studies have primarily evaluated NEC, feeding tolerance, growth, and other short-term neonatal outcomes. Direct comparative evidence regarding long-term neurodevelopment remains limited. Although available observational studies have reported favorable neurodevelopmental outcomes associated with an EHMD, these findings require confirmation in prospective randomized studies [37,38]. Accordingly, evidence concerning an EHMD should be considered separately from evidence concerning MOM, DHM, or HMDF alone.

### 3.4. Importance of Exposure Definition

The source, dose, timing, duration, processing, and fortification of HM may each influence nutritional and clinical outcomes. Furthermore, the comparison group varies across studies: MOM may be compared with formula, DHM with formula, fortified with unfortified HM, or an EHMD with a diet containing one or more cow’s milk-derived products. These comparisons address different clinical questions and should not be combined without qualification [35,36,39].

## 4. Human Milk Exposure and Neurodevelopment

Representative clinical studies illustrating the relationships among HM source, nutrient adequacy, fortification strategy, growth, and neurodevelopment are summarized in Table 2. The table highlights both the principal findings and key methodological limitations to facilitate interpretation according to evidentiary strength.

HM provides nutrients and bioactive components that are biologically relevant to brain development, including long-chain polyunsaturated fatty acids (LCPUFAs), cholesterol, phospholipids, HMOs, lactoferrin, growth factors, and micronutrients. These components may support neuronal membrane formation, myelination, synaptogenesis, vascular development, and regulation of inflammatory and oxidative pathways. Nevertheless, biological plausibility should be distinguished from clinical evidence. Most studies examining HM exposure and neurodevelopment in preterm infants are observational, and relatively few have evaluated specific milk sources or fortification strategies.

### 4.1. Observational Evidence for Neurodevelopmental Outcomes

Several longitudinal cohort studies have reported associations between greater HM exposure during neonatal hospitalization and more favorable cognitive, language, or motor outcomes in infants born very or extremely preterm. In studies of extremely low-birth-weight infants, greater daily and cumulative exposure to HM in the NICU was associated with higher developmental scores at 18 and 30 months’ corrected age [28,29,30]. Other cohorts have reported that these associations may persist into school age, particularly for measures of cognition, working memory, academic achievement, and motor performance [49].

Some studies have also suggested a dose–response relationship, with greater duration or volume of HM exposure associated with more favorable developmental outcomes [29,30,50]. Dose–response findings strengthen the possibility of a biological relationship but do not eliminate confounding. Infants receiving larger amounts of MOM may differ systematically from those receiving less or no MOM with respect to maternal education, socioeconomic status, maternal health, parental involvement, access to lactation support, and severity of neonatal illness [51]. Although many studies adjust for several of these variables, residual and unmeasured confounding cannot be excluded.

The source of HM is also important. Most studies reporting favorable neurodevelopmental associations have evaluated MOM or have combined MOM and DHM into a single exposure category [35]. Evidence specifically examining DHM and long-term neurodevelopment is considerably more limited [35]. Randomized comparisons of DHM and preterm formula have primarily focused on NEC, feeding tolerance, and growth rather than neurodevelopment [35,52]. Available follow-up studies have not consistently demonstrated a clear neurodevelopmental advantage of DHM over formula [35,40,41]. Thus, the neurodevelopmental associations reported for MOM should not be assumed to apply equally to pasteurized DHM.

### 4.2. Human Milk Exposure and Brain Imaging

Neuroimaging studies provide an intermediate outcome between neonatal nutritional exposure and later functional development. Greater exposure to HM during neonatal hospitalization has been associated with larger total and regional brain volumes, greater white matter volume, improved white matter microstructural organization, and more mature functional connectivity at term-equivalent age or later childhood [27,49,53,54,55]. Some studies have also reported associations with larger deep nuclear gray matter or hippocampal volumes, although findings have not been uniform across cohorts.

These imaging findings are biologically plausible because the period between very preterm birth and term-equivalent age is characterized by rapid brain growth, oligodendrocyte maturation, synaptogenesis, and myelination [56]. Nutrient deficiencies, systemic inflammation, infection, and impaired somatic growth during this period may affect structural and functional brain development [43,57,58]. HM exposure could influence these processes through nutrient provision, bioactive components, or reduction in inflammatory neonatal morbidities [27,59,60].

However, brain imaging outcomes should not be regarded as interchangeable with long-term neurodevelopment [61,62]. Differences in imaging modality, timing, brain regions examined, exposure definitions, and adjustment for clinical confounders limit comparison among studies [62]. In addition, associations between HM exposure and brain volume do not establish that HM directly caused the observed structural differences [27,59,61]. Brain imaging therefore provides supportive evidence of a potential pathway but not definitive evidence of clinical benefit.

### 4.3. Bioactive Components and Biological Plausibility

Several constituents of HM have potential roles in neurological development. Docosahexaenoic acid (DHA) contributes to neuronal membrane structure, retinal development, and myelination [4,5,27]. Gangliosides and milk fat globule membrane-derived lipids participate in neuronal signaling and membrane organization [10,13]. HMOs may influence neurodevelopment indirectly through the gut microbiome and immune signaling, while lactoferrin has immunological and potentially neurotrophic properties [11,12,34]. Micronutrients, including vitamin B6 and carotenoids, and neurotrophic factors such as brain-derived neurotrophic factor and S100B may also contribute to early neurological development [14,15].

Much of the evidence concerning individual bioactive components is derived from mechanistic, animal, term-infant, or formula-supplementation studies rather than clinical studies of preterm infants receiving HM [33,34,63]. Furthermore, concentrations of these components vary according to gestational age at delivery, stage of lactation, maternal characteristics, milk storage, pasteurization, and processing. It is therefore not possible to infer the neurodevelopmental effect of HM solely from the presence of individual biologically active components [14,63]. These data support biological plausibility but require confirmation in well-designed clinical studies [14,15].

### 4.4. Null and Inconsistent Findings

Not all studies have demonstrated an independent association between HM exposure and neurodevelopment. In some cohorts, initially favorable associations were attenuated or no longer statistically significant after adjustment for maternal education, socioeconomic factors, neonatal illness, or post-discharge environment [51,64]. Other studies have reported benefits in selected domains, such as cognition or motor function, but not in language, behavior, or global developmental scores [28,37]. Differences in gestational age, milk dose, timing of exposure, fortification practices, follow-up age, and assessment instruments may partly explain these inconsistencies [35].

There is also limited evidence from randomized trials. Random assignment to MOM is neither feasible nor ethical [59], and trials comparing DHM with formula or different fortification strategies have generally not been powered for long-term neurodevelopmental outcomes. Consequently, the available literature cannot definitively determine whether HM exposure itself improves neurodevelopment or whether it acts as one component of a broader combination of nutritional, clinical, maternal, and socioeconomic influences.

### 4.5. Interpretation of the Evidence

Overall, greater exposure to HM, particularly MOM, during neonatal hospitalization is associated with more favorable neurodevelopmental and neuroimaging outcomes in many, but not all, observational studies [28,30,50]. The consistency of some findings, reported dose–response relationships, and biological plausibility support the possibility of a developmental benefit. However, causal inference remains limited by residual confounding, heterogeneous exposure definitions, variable fortification practices, and the scarcity of randomized evidence [35,50,64,65].

The current evidence therefore supports promotion of MOM as the preferred source of nutrition for very preterm infants, consistent with existing clinical guidelines [65,66], but does not justify treating MOM, DHM, HMDF, and an EHMD as equivalent interventions with respect to neurodevelopmental outcomes [35,65]. Further studies should quantify the source, dose, timing, duration, and nutrient composition of HM exposure and should incorporate standardized neurodevelopmental assessment together with brain imaging and relevant maternal and socioeconomic covariates [28,35,59].

## 5. Protein Adequacy, Route of Nutrient Delivery, and Neurodevelopment

As survival of VPIs has improved, long-term neurodevelopment has become an increasingly important outcome of neonatal care. In Japan, survival among infants born at 22–27 weeks’ gestation improved substantially between 2005 and 2015, whereas comparable improvement in neurodevelopmental outcomes was not consistently observed [42]. Nutrition is a potentially modifiable component of neonatal care, but the relationship between prescribed nutrient intake and later development is complex and depends on nutrient adequacy, route of delivery, feeding tolerance, illness severity, and postnatal growth.

### 5.1. Route and Adequacy of Nutrient Delivery

Associations between early protein or energy intake and neurodevelopment do not necessarily imply that simply increasing nutrient provision is associated with developmental outcomes. Observational studies have linked higher early nutrient intake, particularly enteral intake, with more favorable developmental outcomes [42,43,58,67,68]. However, infants who tolerate earlier and greater enteral feeding are often less severely ill, making it difficult to separate the effect of nutrition from the effect of clinical stability.

Randomized evidence provides a more cautious perspective. A randomized trial of enhanced early parenteral nutrition did not demonstrate improvement in later neurodevelopmental outcomes [44]. This null finding suggests that increasing nutrient delivery through the parenteral route does not necessarily translate into functional benefit. It may also indicate that the timing, route, balance, and biological context of nutrient delivery are important and that developmental outcomes cannot be predicted from prescribed protein and energy intake alone [69,70].

Prolonged dependence on parenteral nutrition has been associated with less favorable growth and brain imaging findings in observational studies [71,72]. Nevertheless, this association may reflect illness severity, feeding intolerance, infection, surgery, or other complications rather than a direct adverse effect of parenteral nutrition. Parenteral nutrition remains essential when adequate enteral intake cannot be achieved and should not be interpreted as intrinsically detrimental to neurodevelopment [70].

### 5.2. Interpreting Nutritional and Neurodevelopmental Evidence

Assessment of neurodevelopment after preterm birth presents several methodological challenges. Outcomes vary according to the age at evaluation and the instrument used, and developmental scores in early childhood do not always predict later executive function, academic achievement, or behavior [28,73]. Attrition during follow-up may introduce selection bias, while differences in neonatal illness, family environment, early intervention, and post-discharge nutrition may further influence outcomes [51,64,70].

The available evidence also does not identify a single HM exposure threshold that reliably improves neurodevelopment [35,50,59]. Studies have variously evaluated any HM, predominant HM, exclusive HM, daily dose, cumulative dose, and duration of exposure [30,35,50]. In addition, many reports do not adequately describe the proportions of MOM and DHM, milk composition, fortifier source, timing of fortification, or actual protein and energy intake [35,59]. These differences limit direct comparison and synthesis.

Overall, greater exposure to MOM during neonatal hospitalization is associated with more favorable neurodevelopmental outcomes in many, but not all, observational studies [28,30,50]. Evidence specifically supporting a long-term neurodevelopmental benefit of DHM is less certain, and randomized evidence is scarce. The current literature therefore supports MOM as the preferred nutritional source for VPIs but does not establish that HM exposure alone causes improved neurodevelopment [28,35,59,65]. Future studies should quantify milk source, dose, timing, duration, nutrient composition, and fortification and should incorporate long-term, domain-specific developmental assessments together with relevant clinical, maternal, and socioeconomic covariates [28,35,59,70].

## 6. Human Milk Fortification Strategies in VPIs

Unfortified HM generally does not provide sufficient protein, energy, minerals, and micronutrients to meet the estimated requirements of very preterm infants (VPIs) at customary feeding volumes [74,75,76]. Fortification is therefore required to support postnatal growth and nutrient accretion [74,76]. However, the established need to fortify HM should be distinguished from the question of which fortifier source or fortification strategy provides the greatest clinical or neurodevelopmental benefit [77,78].

Fortifiers used in neonatal care include cow’s milk-derived fortifiers (CMDFs) and HM-derived fortifiers (HMDFs). CMDFs are widely available and have an established role in increasing the nutrient content of mother’s own milk (MOM) and donor human milk (DHM) [74,76]. HMDFs also increase protein and energy delivery while allowing avoidance of bovine-derived products as part of an exclusive human milk diet (EHMD) [36,52,78]. These approaches differ in composition, processing, availability, cost, and supporting evidence and should therefore be evaluated separately.

### 6.1. Cow’s Milk-Derived and Human Milk-Derived Fortifiers

Both CMDFs and HMDFs are intended to address the nutrient limitations of unfortified HM. Most clinical studies support fortification in general for improving short-term growth, but direct comparisons between CMDFs and HMDFs remain relatively limited. Moreover, studies differ in the base milk provided, timing and dose of fortification, use of formula, feeding protocols, and characteristics of the infants enrolled.

Mechanistic studies have suggested that exposure to bovine-derived proteins may influence intestinal inflammatory or oxidative pathways in preterm infants [45,52,79]. Other laboratory studies have reported differences between CMDF and HMDF in their effects on oxidative activity, milk fat globule structure, or selected bioactive properties of HM [80,81,82]. These findings provide biological plausibility for differences between fortifier sources but should not be interpreted as demonstrating superiority in clinical or neurodevelopmental outcomes.

CMDFs have been used successfully in large numbers of preterm infants and provide protein, minerals, and micronutrients required for growth. Most infants tolerate CMDF without clinically recognized adverse feeding events. In a nationwide Japanese cohort of 2,476 infants with complete data who received DHM followed by CMDF, 315 infants (12.7%) experienced documented adverse feeding events, most commonly abdominal distension, increased gastric residuals, vomiting, and suspected cow’s milk protein allergy. NEC was uncommon, occurring in only six infants. After multivariable adjustment, delayed achievement of an enteral feeding volume of 100 mL/kg/day was the only factor independently associated with adverse feeding events, whereas routinely available clinical variables had limited discriminatory ability (area under the curve, 0.611) [47]. These findings indicate that CMDF is tolerated by most preterm infants but also suggest that infants with delayed enteral feeding progression may require closer monitoring and individualized consideration of alternative fortification strategies. Because this study was retrospective and the timing of CMDF initiation was not directly available, the findings should be considered hypothesis-generating rather than evidence that CMDF caused the reported events.

In a secondary subgroup analysis of a multicenter randomized trial, exposure to a CMDF within an otherwise HM-based diet was associated with higher risks of NEC and the composite outcome of NEC surgery or death than a diet using an HMDF [45]. Lower head circumference growth was also reported in the CMDF group [45]. Because these findings were derived from a subgroup analysis and neurodevelopment was not directly assessed, they should be considered hypothesis-generating rather than definitive evidence of a neurodevelopmental effect.

### 6.2. Exclusive Human Milk Diet and Short-Term Outcomes

An EHMD generally consists of MOM, supplemented when necessary with DHM, together with an HMDF and avoidance of cow’s milk-derived formula and fortifier [36,52,78]. An EHMD is therefore a multicomponent feeding strategy rather than an intervention involving only a particular fortifier. Outcomes attributed to an EHMD may reflect differences in formula exposure, proportions of MOM and DHM, feeding advancement, nutrient delivery, fortification practices, or other aspects of clinical care [35,59,78].

Randomized trials and observational studies have evaluated the effects of an EHMD on NEC, feeding tolerance, growth, late-onset sepsis, bronchopulmonary dysplasia, and retinopathy of prematurity [36,39,83,84,85,86]. Some studies have reported lower rates of NEC or other neonatal morbidities with an EHMD than with diets containing cow’s milk-derived products [36,52]. However, the results have not been uniform across studies, and the certainty of evidence varies by outcome [35,78]. Many reports are retrospective, involve relatively small cohorts, or compare bundled feeding protocols rather than fortifier source alone [35,59,78].

A multicenter phase III randomized trial conducted in Japan compared an EHMD with a standard feeding strategy containing cow’s milk-derived products in very low-birth-weight infants [46]. The EHMD group had greater weight gain velocity and achieved full enteral feeding earlier, without evidence of an increase in serious treatment-related adverse events [46]. This trial supports the short-term growth efficacy and clinical feasibility of the evaluated EHMD protocol in the studied population. However, the trial was open-label, NEC occurred infrequently, and neurodevelopmental outcomes were not evaluated. Its findings therefore should not be interpreted as demonstrating prevention of NEC or improvement in long-term neurodevelopment.

### 6.3. Fortification and Neurodevelopmental Outcomes

Direct evidence comparing the effects of CMDF and HMDF on long-term neurodevelopment is limited. Observational studies of infants receiving an EHMD have generally reported reassuring developmental outcomes. One cohort study found a lower adjusted risk of motor impairment at 3 years among infants receiving an EHMD than among those receiving a diet containing bovine-derived products (odds ratio 0.74, 95% confidence interval 0.56–0.98) [37]. Other studies have reported developmental scores within expected ranges or higher cognitive scores following an EHMD [38,87].

These findings should be interpreted cautiously. The available studies are predominantly observational, and differences in illness severity, access to MOM, socioeconomic factors, clinical practices, and follow-up may influence the results [37,51]. Some studies have lacked a contemporaneous comparator group or have been insufficiently powered to assess individual developmental domains [35,37]. Furthermore, an association between an EHMD and neurodevelopment cannot establish that the HMDF component was responsible for the observed outcome [35,88].

At present, the evidence supports the nutritional necessity of fortifying HM but does not establish that HMDF is associated with long-term neurodevelopment compared with CMDF. Potential effects of fortifier source on neurodevelopment may be mediated through nutrient delivery, growth, body composition, feeding tolerance, or neonatal morbidity, but these pathways have not been adequately tested. Long-term follow-up of randomized trials using standardized developmental assessments is needed.

### 6.4. Timing of Fortification

The optimal timing of HM fortification remains uncertain and varies among institutions. Fortification is often initiated when enteral feeding reaches approximately 50–100 mL/kg/day, although earlier introduction has been studied [48,76]. In a randomized trial of extremely preterm infants, fortification initiated as early as the second postnatal day did not increase feeding intolerance or NEC compared with later fortification [48]. However, the study was not designed to establish an effect on long-term neurodevelopment.

Earlier fortification may reduce cumulative protein and mineral deficits, particularly in infants receiving predominantly DHM, infants with early growth faltering, and those whose total fluid intake is restricted [75,76]. Conversely, the timing of fortification must account for clinical stability, feeding tolerance, milk volume, nutrient composition, and the osmolality and composition of the fortified milk [74,77]. Current evidence does not support a single initiation threshold that is appropriate for all VPIs [48,76].

In the Japanese randomized trial, HMDF was initiated when enteral feeding reached approximately 50 mL/kg/day, and feeding volumes were subsequently advanced according to tolerance [46]. This represents one protocol evaluated within a specific randomized trial rather than a universally established standard. It provides a clinically relevant example of structured fortification, but its applicability to other products, populations, and feeding protocols requires further evaluation (Figure 1).

### 6.5. Standard, Adjustable, and Targeted Fortification

Fortification can be provided using standard, adjustable, or targeted approaches. Standard fortification adds a fixed quantity of fortifier based on an assumed HM composition. Because the protein and energy content of MOM and DHM varies substantially, standard fortification may result in nutrient delivery that is lower or higher than intended [89,90].

Adjustable fortification modifies protein supplementation according to growth and biochemical indices, commonly including blood urea nitrogen [74,75]. Targeted fortification uses measured milk macronutrient content to individualize protein, energy, or fat supplementation [90,91]. These approaches may improve the accuracy of nutrient delivery, but evidence demonstrating superiority in long-term neurodevelopmental outcomes remains insufficient [89,91].

A stepwise approach may begin with a protein intake appropriate for the infant’s weight and clinical condition, with subsequent adjustment according to growth velocity, linear and head circumference growth, energy intake, blood urea nitrogen, electrolyte balance, acid–base status, and feeding tolerance [77,91]. Recommended protein intake should be regarded as a range rather than a fixed target; increasing protein without sufficient energy or without monitoring metabolic tolerance may not improve tissue accretion [75,89].

In infants requiring fluid restriction, achieving adequate protein and energy intake may require greater nutrient density rather than increasing feeding volume. A modified fortification strategy for such infants, adapted from the feeding protocol evaluated in the Japanese randomized trial, is illustrated in Figure 2.

### 6.6. Clinical Interpretation and Research Priorities

Fortification of HM is necessary for most VPIs, but current evidence does not support treating all fortifiers or fortification protocols as equivalent. It also does not establish that a particular fortifier source is independently associated with neurodevelopment [78,89]. Selection of a fortification strategy should consider the available milk source, estimated nutrient intake, infant size and maturity, growth trajectory, fluid allowance, metabolic tolerance, product availability, and local clinical experience [76,77].

Future trials should separate the effects of base milk, fortifier source, timing, nutrient dose, and avoidance of formula. They should report actual protein and energy intake and include body composition, brain imaging, and standardized long-term neurodevelopmental outcomes. Comparative effectiveness, safety, feasibility, and cost should also be evaluated because these factors influence the applicability of fortification strategies across neonatal care settings [78,89].

Practical implementation also requires consideration of donor human milk and HMDF availability, product cost, milk-bank infrastructure, local feeding practices, and healthcare resources. Access to DHM and HMDF varies substantially among countries and institutions, which may limit the feasibility and generalizability of an EHMD. Consequently, nutritional strategies should be individualized not only according to infant-specific nutritional needs but also within the resources and infrastructure of the local healthcare setting.

## 7. Indirect Pathways Linking Human Milk Exposure to Neurodevelopment

Neurodevelopmental outcomes after very preterm birth are influenced not only by nutrient intake but also by neonatal morbidities occurring during a critical period of brain development. NEC, LOS, BPD, and severe ROP have each been associated with an increased risk of later neurodevelopmental impairment. HM exposure may therefore be related to neurodevelopment through both direct nutritional pathways and indirect pathways involving a lower burden of neonatal morbidity. However, evidence supporting these indirect pathways is derived predominantly from observational studies and should not be interpreted as demonstrating causality.

### 7.1. Intestinal Integrity, NEC, and Systemic Inflammation

Among the short-term outcomes associated with HM feeding, the evidence is most consistent for a lower risk of NEC. Cohort studies and meta-analyses have reported dose–response associations between greater exposure to HM, particularly MOM, and lower risks of NEC, LOS, and mortality [16,17,18,83,92,93]. HM contains oligosaccharides, lactoferrin, secretory immunoglobulin A, epidermal growth factor, and other bioactive factors that may support epithelial barrier maturation, microbial homeostasis, and regulation of inflammatory signaling [94,95,96,97,98,99]. These mechanistic findings provide biological plausibility but do not, by themselves, establish that individual milk components are associated with neurodevelopmental outcomes.

Severe NEC can be accompanied by systemic inflammation, surgery, prolonged nutritional interruption, and impaired postnatal growth, all of which may adversely affect the developing brain [56]. Accordingly, prevention of NEC represents one plausible indirect pathway linking HM exposure with later neurodevelopment [60]. Nevertheless, infants receiving greater amounts of MOM may differ from infants receiving less MOM in maternal, socioeconomic, and clinical characteristics [51,64]. Residual confounding therefore remains an important limitation of observational studies.

### 7.2. Infection, Pulmonary Morbidity, and Retinal Vascular Development

Predominant or greater HM exposure has also been associated with lower rates of LOS, BPD, and ROP in several cohort studies and meta-analyses [19,20,21,23,24,33,100,101,102]. The strength and consistency of these associations vary according to the outcome, milk source, exposure definition, and study design. In particular, findings for MOM should not automatically be extrapolated to pasteurized DHM, and findings for an EHMD should not be attributed solely to the use of a HMDF.

Potential mechanisms include reduced intestinal permeability and bacterial translocation, attenuation of systemic inflammation and oxidative stress, and provision of nutrients involved in neural and vascular development. LCPUFA, particularly DHA, contribute to neuronal membrane formation and retinal development, whereas postnatal insulin-like growth factor-1 deficiency has been associated with impaired vascular development in both the lung and retina [6,7,8,103,104,105]. These pathways are biologically plausible, but direct evidence that HM is associated with neurodevelopment by modifying these specific pathways remains limited.

LOS and BPD may expose preterm infants to prolonged inflammation, respiratory support, corticosteroids, sedative medications, and nutritional deficits [106]. Severe ROP may also indicate disrupted vascular and growth-factor signaling during a period of rapid brain development [9]. Reducing these morbidities could therefore contribute indirectly to more favorable developmental trajectories. However, most studies have evaluated neonatal morbidity rather than neurodevelopment as the primary outcome, and mediation through these morbidities has rarely been formally tested [106].

### 7.3. Interpretation of the Multi-Organ Evidence

Taken together, the available evidence supports a conceptual model in which HM exposure may be associated with neurodevelopment through several interconnected pathways, including nutrient delivery, intestinal maturation, immune regulation, growth, and a lower burden of neonatal morbidity [59,61]. The evidence is strongest for associations between HM exposure and selected short-term outcomes, particularly NEC, whereas evidence directly linking these effects to later neurodevelopment is less certain [16,36]. HM should therefore be regarded as a heterogeneous nutritional exposure whose effects depend on milk source, dose, timing, processing, fortification, and the clinical characteristics of the infant [59,78]. Further prospective studies are needed to determine whether reductions in neonatal morbidity mediate the relationship between specific HM-based feeding strategies and long-term neurodevelopment.

## 8. Conclusions

HM plays a central role in the nutritional management of VPIs. The evidence is strongest for associations between HM exposure and selected short-term outcomes, particularly NEC, while associations with BPD, ROP, LOS, growth, and neurodevelopment vary according to milk source, dose, study design, and clinical context. Greater exposure to MOM is associated with more favorable neurodevelopmental and neuroimaging outcomes in many observational studies, although causal inference remains limited.

Nutritional adequacy is also critical. Unfortified HM generally does not meet the protein, energy, mineral, and micronutrient requirements of VPIs, making fortification necessary for most infants. Current evidence supports individualized attention to protein and energy delivery, growth, feeding tolerance, and metabolic status, but does not establish that a particular fortifier source independently improves long-term neurodevelopment.

Taken together, available evidence supports an integrated approach based on early and sustained HM exposure, preferential use of MOM, progressive enteral feeding, adequate nutrient delivery, and appropriate fortification. Within the limitations of current evidence, an EHMD represents a biologically plausible and clinically promising strategy for providing adequate early nutrition while avoiding bovine-derived products during a vulnerable period of development. However, whether the short-term nutritional and clinical advantages associated with an EHMD translate into sustained neurodevelopmental benefits remains to be established.

Future studies should clearly define milk source, dose, timing, duration, processing, fortifier source, and actual nutrient intake and should incorporate standardized measures of growth, body composition, brain imaging, and long-term, domain-specific neurodevelopmental outcomes. Where feasible, adequately powered randomized trials are needed to evaluate specific modifiable nutritional interventions, including fortification strategies and nutrient delivery. These should be complemented by well-designed prospective cohort studies to examine exposures that cannot readily or ethically be randomized, such as MOM availability and cumulative human milk exposure. Long-term follow-up of existing and future trials will be essential to determine whether short-term improvements in nutrient delivery, growth, feeding tolerance, or neonatal morbidity translate into sustained neurodevelopmental benefits. These priorities will help refine individualized nutritional strategies that preserve the biological advantages of HM while meeting the nutritional requirements of VPIs across diverse clinical settings.

## Figures and Tables

**Figure 1:**
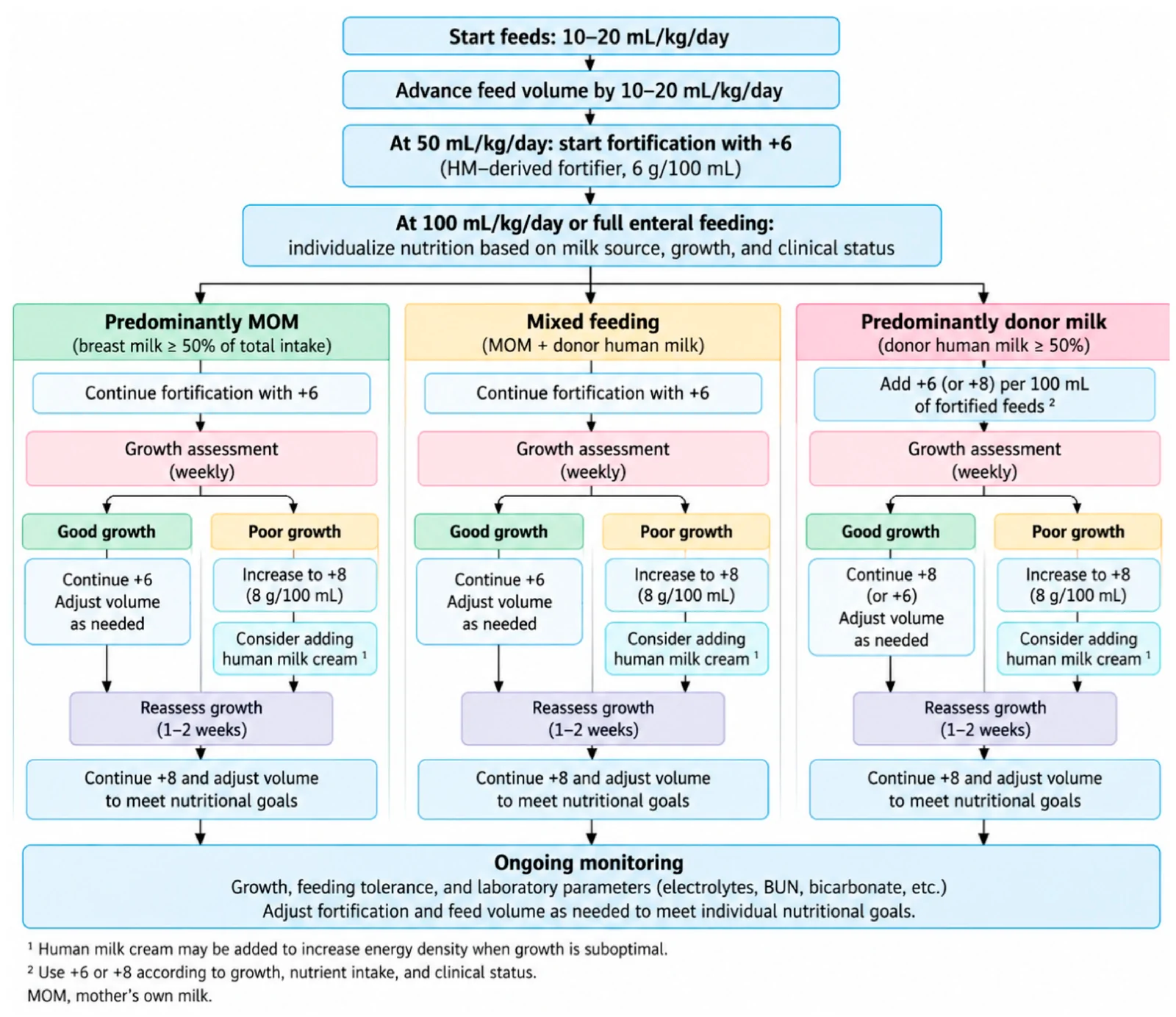
**Feeding protocol for VPIs using human milk-derived fortification (Based on JASMINE trial protocol).** Conceptual Framework for Human Milk-Derived Fortification in Very Preterm Infants. Conceptual representation of the stepwise fortification strategy evaluated in the Japanese multicenter randomized trial of an exclusive human milk diet (EHMD). Human milk-derived fortification was initiated when enteral feeding reached approximately 50 mL/kg/day using a standard fortifier (+6), followed by progressive advancement according to feeding tolerance, growth response, gestational age, and clinical condition. In the trial, the evaluated EHMD protocol was associated with greater weight gain velocity and earlier achievement of full enteral feeding without evidence of an increase in serious treatment-related adverse events [46]. This framework illustrates the feeding and fortification approach evaluated in the trial and should not be interpreted as a universally applicable clinical protocol.

**Figure 2:**
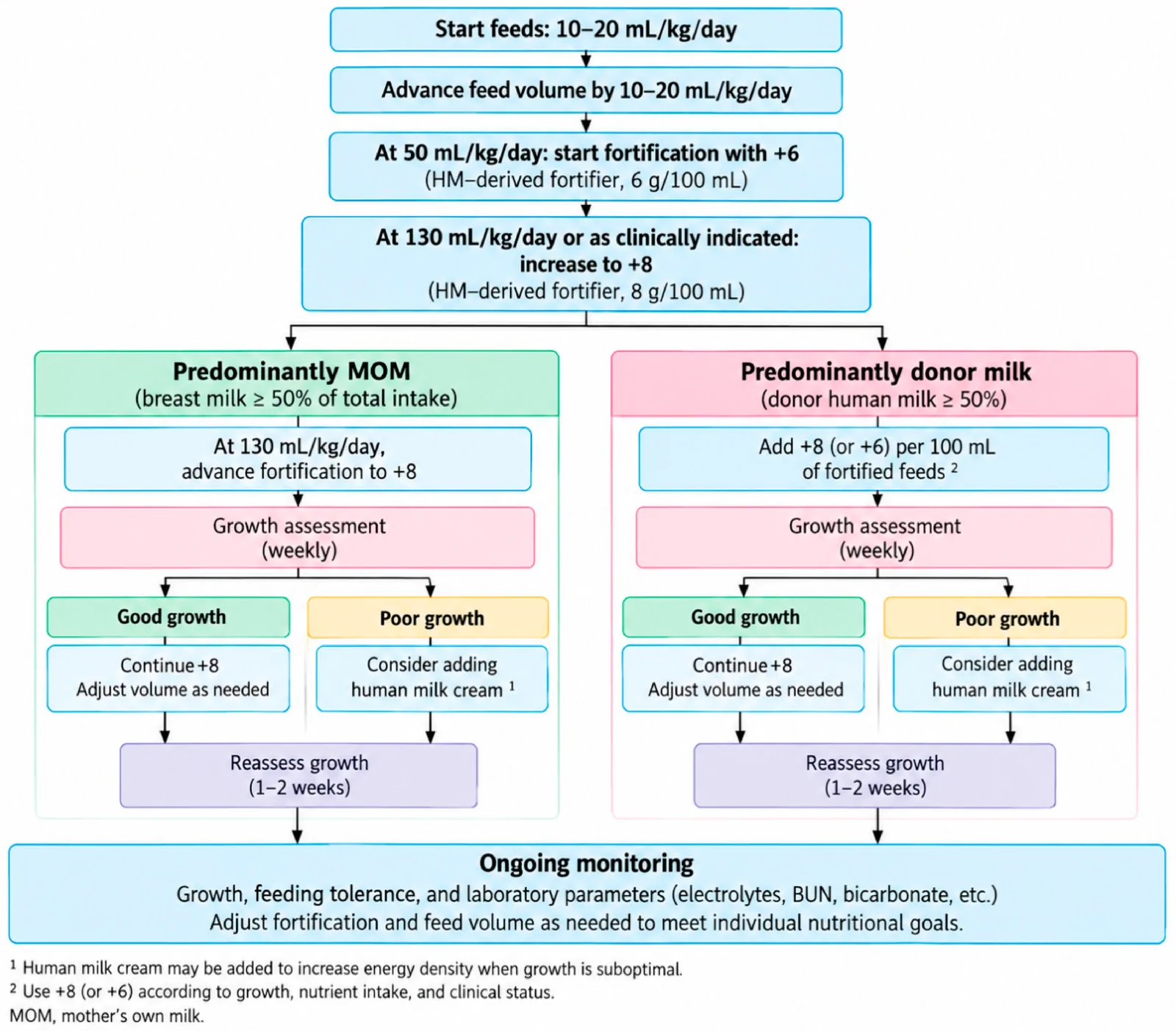
**Modified feeding protocol for fluid-restricted infants (Based on JASMINE trial protocol).** Conceptual Framework for Individualized Fortification in Fluid-Restricted Very Preterm Infants. Conceptual representation of an individualized fortification approach for very preterm infants (VPIs) requiring fluid restriction. This framework illustrates how nutrient density may be adjusted when feeding volume is limited and should not be interpreted as a universally applicable clinical protocol. A higher-protein fortifier (+8) may be used to increase nutrient density when feeding volumes are constrained, with the degree of fortification individualized according to measured or estimated nutrient intake, growth, biochemical indices, metabolic tolerance, and clinical condition. This approach may be considered in infants with bronchopulmonary dysplasia, congenital heart disease, or other conditions requiring fluid restriction. Its effects on growth, body composition, head growth, and long-term neurodevelopment require further evaluation.

**Table 1:** Bioactive Components of Human Milk and Their Roles in Neurodevelopment.

Component	Role in Neurodevelopment
Long-chain polyunsaturated fatty acids (LCPUFAs), particularly docosahexaenoic acid (DHA)	contribute to neuronal membrane structure, retinal development, and myelination [4,5]
Growth factors, including insulin-like growth factor-1 (IGF-1)	participate in growth and vascular development; postnatal IGF-1 deficiency has been associated with impaired vascular development in the lung and retina [6,7,8,9]
Gangliosides	participate in neuronal membrane organization and signaling and may contribute to early brain development [10]
Human milk oligosaccharides (HMOs) and lactoferrin	may influence neurodevelopment indirectly through microbiome and immune signaling; combined effects remain an area of emerging mechanistic research [11,12]
Milk fat globule membrane (MFGM)-derived lipids	participate in membrane organization, myelination, and neuronal signaling [13]
Micronutrients, including vitamin B6 and carotenoids	have been associated with early neurobehavioral development, although evidence varies by nutrient and study design [14]
Neurotrophic factors, including brain-derived neurotrophic factor (BDNF) and S100B	are biologically relevant to neuronal survival and synaptic development; clinical evidence in preterm infants remains limited [15]

**Table 2:** Representative Clinical Evidence Linking Human Milk Exposure, Nutritional Adequacy, Fortification, and Neurodevelopment in Preterm Infants.

Study [Ref.]	Design/Population	Nutritional Exposure	Main Outcome	Major Finding	Key Limitation	Clinical Interpretation
Vohr et al., 2006 [29]	Prospective cohort; ELBW infants	HM dose during NICU hospitalization	Neurodevelopment at 18 months	Greater HM intake was associated with more favorable developmental outcomes	Observational exposure; residual maternal and socioeconomic confounding	Supports a dose–response association between early HM exposure and development, but does not establish causality
Vohr et al., 2007 [30]	Longitudinal follow-up; ELBW infants	Cumulative HM exposure	Neurodevelopment at 30 months	Favorable associations with HM exposure persisted at 30 months	Observational analysis; HM source and nutritional composition not fully characterized	Suggests persistence of the association between neonatal HM exposure and later development
Belfort et al., 2016 [28]	Prospective longitudinal cohort; infants <30 weeks GA	Predominantly MOM/HM exposure	Neurocognition and brain MRI at 7 years	Greater HM exposure was associated with favorable neurocognitive and neuroimaging measures	Nonrandomized HM exposure; potential residual confounding	Links early HM exposure with both structural brain measures and later function, while remaining associative
O’Connor et al., 2016 [40]	Multicenter randomized trial; VLBW infants	Supplemental DHM vs. preterm formula when MOM insufficient	Bayley-III at 18 months	Supplemental DHM did not improve cognitive, language, or motor scores compared with formula	High background MOM exposure; not a comparison of MOM vs. formula	Demonstrates that neurodevelopmental findings for MOM should not automatically be extrapolated to pasteurized DHM
Colaizy et al., 2024 [41]	Randomized clinical trial follow-up; extremely preterm infants	DHM vs. preterm formula	Long-term neurodevelopment	No clear neurodevelopmental advantage of DHM over preterm formula	Intervention addresses supplemental feeding rather than MOM exposure	Reinforces distinction between evidence for MOM and evidence for DHM
Stephens et al., 2009 [42]	Observational cohort; ELBW infants	First-week protein and energy intake	18-month neurodevelopment	Higher early protein and energy intakes were associated with more favorable developmental outcomes	Nutrient intake strongly related to illness severity and feeding tolerance	Supports nutritional adequacy as a potential contributor to development, but causality remains uncertain
Schneider et al., 2018 [43]	Prospective observational study; preterm infants	Early nutrient intake	MRI brain growth	Higher nutrient intake during the first 2 weeks was associated with measures of brain growth	Observational; prescribed/intake exposure may reflect clinical stability	Provides an intermediate link between early nutritional adequacy and structural brain development
Morris et al., 2022 [44]	Randomized controlled trial; preterm infants	Enhanced early parenteral nutrition	Later neurodevelopment	Enhanced parenteral nutrient provision did not improve neurodevelopment	Route and composition differ from enteral HM-based nutrition	Shows that increasing nutrient provision alone does not necessarily translate into neurodevelopmental benefit
Lucas et al., 2020 [45]	Secondary subgroup analysis of multicenter randomized trial	CMDF within HM-based diet vs. HMDF/EHMD strategy	NEC, NEC surgery/death, head growth	CMDF exposure was associated with less favorable selected short-term outcomes and head growth	Secondary subgroup analysis; neurodevelopment not directly assessed	Hypothesis-generating evidence for differences between fortifier sources; not proof of long-term developmental benefit
Chou et al., 2025 [37]	Observational cohort	EHMD vs. diet containing bovine-derived products	Neurodevelopment at 3 years	EHMD was associated with a lower adjusted risk of motor impairment	Observational design; potential differences in clinical practice and patient selection	Provides a favorable long-term developmental signal requiring confirmation in randomized studies
Hair et al., 2022 [38]	Multicenter observational study	EHMD vs. mixed HM + bovine milk-based diet	Neurodevelopment	Favorable/reassuring developmental outcomes were reported with EHMD	Nonrandomized exposure; bundled nutritional strategy	Supports further investigation of EHMD but cannot isolate the independent effect of HMDF
Mizuno et al., 2026 (JASMINE) [46]	Multicenter phase III randomized trial; VLBW infants	EHMD vs. standard feeding strategy containing cow’s milk-derived products	Growth, full enteral feeding, safety	EHMD improved weight-gain velocity and shortened time to full enteral feeding without an increase in serious treatment-related adverse events	Open-label; NEC infrequent; long-term neurodevelopment not evaluated	Provides randomized evidence for short-term growth efficacy and feasibility of the evaluated EHMD protocol, but not for long-term neurodevelopment
Tani & Mizuno, 2026 [47]	Nationwide retrospective HMB cohort	DHM followed by CMDF	Adverse feeding events	Most infants tolerated CMDF; delayed attainment of 100 mL/kg/day was independently associated with adverse feeding events	Retrospective; timing of CMDF initiation unavailable; limited discrimination	Supports individualized monitoring during fortification rather than attributing feeding intolerance directly to CMDF
Salas et al., 2023 [48]	Randomized trial; extremely preterm infants	Early vs. later HM fortification	Feeding tolerance, NEC, nutritional outcomes	Fortification from the early postnatal period did not increase feeding intolerance or NEC	Not designed/powered for long-term neurodevelopment	Supports feasibility of earlier fortification but does not define an optimal universal threshold

## Data Availability

No new data were created or analyzed in this study. Data sharing is not applicable to this article.
